# Impact of the fermentation parameters pH and temperature on stress resilience of *Lactobacillus reuteri* DSM 17938

**DOI:** 10.1186/s13568-019-0789-2

**Published:** 2019-05-17

**Authors:** Armando Hernández, Christer U. Larsson, Radoslaw Sawicki, Ed W. J. van Niel, Stefan Roos, Sebastian Håkansson

**Affiliations:** 1 Department of Molecular Sciences, Swedish University of Agricultural Sciences, PO Box 7015, 750 07 Uppsala, Sweden; 2 Center for Applied Life Sciences, Applied Microbiology, Lund University, Box 124, 221 00 Lund, Sweden; 3 Present Address: Q-linea AB, Dag Hammarskjölds väg 52A, 752 37 Uppsala, Sweden

**Keywords:** Probiotics, Fermentation technologies, *Lactobacillus reuteri* DSM 17938, Freeze-drying, Bile salt, Survivability

## Abstract

This study was undertaken to investigate the impact of culture pH (4.5–6.5) and temperature (32–37 °C) on the stress resilience of *Lactobacillus reuteri* DSM 17938 during freeze-drying and post freeze-drying exposure to low pH (pH 2) and bile salts. Response-surface methodology analysis revealed that freeze-drying survival rates $\left( {\frac{Ncells\;after\;drying}{Ncells \;before\;drying}\;100} \right)$ were linearly related to pH with the highest survival rate of 80% when cells were cultured at pH 6.5 and the lowest was 40% when cells were cultured at pH 4.5. The analysis further revealed that within the chosen temperature range the culture temperature did not significantly affect the freeze-drying survival rate. However, fermentation at pH 4.5 led to better survival rates when rehydrated cells were exposed to low pH shock or bile salts. Thus, the effect of pH on freeze-drying survival was in contrast to effects on low pH and bile salts stress tolerance. The rationale behind this irreconcilability is based on the responses being dissimilar and are not tuned to each other. Culturing strain DSM 17938 at pH values higher than 5.5 could be a useful option to improve the survivability and increase viable cell numbers in the final freeze-dried product. However, the dissimilar responses for the process- and application parameters tested here suggest that an optimal compromise has to be found in order to obtain the most functional probiotic product possible.

## Introduction

As stated by Rosenstiel and Stange (2010), probiotics are living microorganisms that—if taken in appropriate dosage—may result in a health benefit for the host. The probiotic market has increased in recent years. In 2015, it has exceeded 35 billion USD and is expected to reach revenues of 74 billion USD in 2024 (Grand View Research Inc. 2016). To help realize this expectation, it is essential to better understand how production processes influence product quality. Thus, evaluation of the impact of different production variables on the yield and survivability of the cells in the freeze-dried product is essential to the development of stable probiotics.

Cell stress tolerance is an important issue in the development of stable probiotic products based on freeze-dried formulations of Lactic Acid Bacteria (LAB). Particularly in the case of probiotic bacteria to be ingested, tolerance to low pH in the stomach and bile salts in the intestine is crucial to achieve efficient bioactivity (Yadav and Shukla 2017). Among the different culture variables, temperature, pH, and dissolved oxygen concentration, have previously been shown to be particularly important for the stabilization of freeze-dried bacterial cells of the genera *Lactobacillus* (Schoug et al. 2008; Liu et al. 2014; Béal and Fonseca 2015) and *Bifidobacterium* (Mozzetti et al. 2013).

This have also been shown in preconditioning or physiological condition of bacterial biomass subjected to freeze-drying, where mild abiotic stressors applied during or immediately after the fermentation can improve desiccation tolerance and the stability of dried formulations (Palmfeldt and Hahn-Hägerdal 2000; Liu et al. 2014).

To date, the most studied bacterial genera with probiotic properties are *Lactobacillus* and *Bifidobacterium* (Sánchez et al. 2017). *Lactobacillus reuteri* DSM 17938 is the active ingredient of several probiotic products (e.g. BioGaia Protectis) (Rosander et al. 2008; Mu et al. 2018) and this strain is stabilized by freeze-drying to achieve storage stability of the final product.

Although the fermentation process of the strain DSM 17938 has been well characterized (Burgé et al. 2015; Strömberg et al. 2017; van Niel et al. 2017; Mauro and Garcia 2019), the impact of different fermentation variables on the survival of this strain to freeze-drying and several of the subsequent gastro-intestinal tract (GIT) stress conditions (i.e. low pH and bile salts) has not been deeply explored. In this study, we have evaluated the impact of both culture pH and temperature on the survival rate of strain DSM 17938 under these stress conditions. The results shown here will be of relevance for future development of stable desiccated formulations of *L. reuteri* DSM 17938 with high levels of probiotic activity.

## Materials and methods

### Microorganisms and culture conditions

*Lactobacillus reuteri* DSM 17938 (Rosander et al. 2008), kindly provided by BioGaia AB, was used for all experiments. A previously established and controlled cryopreserved cell bank was used (Garcia et al. 2017) as inoculum throughout this study. In total 12 fermentation batches at 1-L were performed. Each experiment was started from cells retrieved from this cell bank, 10 mL vials containing 10 mL MRS medium (Merck) were inoculated with 100 μL of the cell bank. Each vial was capped and incubated statically for 6 h at 37 °C and the resulting culture was used as the inoculum for the next stage. 1 mL from the 10-mL preculture was inoculated to 500 mL flask containing 500 mL MRS and this culture was incubated statically for 16 h at 37 °C. The fermentations at 1-L scale were performed in 1.8 L Jenny bioreactors (Belach Bioteknik AB). One hundred fifty milliliter from the 500-mL preculture were inoculated into 850 mL MRS medium (pH- and temperature-controlled fermentation) (Strömberg et al. 2017).

The starting pH was 6.5 and the pH control was kept at 4.5, 5.5, or 6.5 using NaOH 3.7 M once the culture reached the set value. The culture temperature was set to either 32 °C or 37 °C according to the planned factorial design. Every batch fermentation was performed for a total of 26 h; thus, all cultures were well in the stationary phase at which cells are better adapted to stressful conditions, which ensures that cells survive better to desiccation (Meng et al. 2008), and the growth was determined by OD_600_ measurement with spectrophotometer Ultrospec 1100 pro.

### Freeze-drying procedure

At the end of each fermentation process, 500 mL of the culture was harvested and centrifuged according to Teixeira et al. (1997) but for 30 min instead of 10 min, at 5000×*g* at 24 °C. The pellet was suspended in 50 mL sucrose 10% (w/v) with a spatula and thoroughly mixed by gentle shaking using a vortex until a homogenous slurry was obtained. One mL of this concentrated cell suspension was dispensed into freeze-drying glass vials (35 vials per batch) and the vials were frozen in an ultrafreezer at − 50 °C for 2 h before being transferred to a Labconco FreeZone^®^ Stoppering Tray Freeze-Dryer. The freeze-drying scheme was set-up as follows: Freezing step: − 40 °C for 3 h; Main drying: − 40 °C for 18 h at 0.2 mbar, − 20 °C for 70 h at 0.2 mbar; secondary drying: 5 °C increments every 2 h up to 20 °C at 0.01 mbar. At the end of the process the vials were capped under vacuum and stored at − 50 °C until further use. For every experimental condition, the freeze-dried cells were rehydrated by triplicate with 1 mL of saline solution (NaCl 9 g/L) for determining the survival rates.

### Viable count

For every sample, serial dilutions were done. For each dilution, 10 µL were plated (Jett et al. 1997) (in triplicate) on MRS agar plates and incubated at 37 °C for 18 h. The resulting colonies were counted and the viability (cfu/mL) value was calculated based on the plated dilution.

### Bile and low pH stress in survival assays

Porcine bile (B8631; Sigma) and bovine bile (B3883; Fluka) were diluted in MRS (Merck) to a final concentration of 0.5% (w/v) and 1% (w/v) bile salts, respectively. Low pH MRS was prepared by adding 1 M HCl to MRS, lowering the pH to pH 2. Freeze-dried cell samples were rehydrated for 20 min with 1 mL saline solution, vortexed and diluted 100 times in PBS (137 mM NaCl, 2.7 mM KCl, 10 mM Na_2_HPO_4_, 1.8 mM KH_2_PO_4_) to reach a target concentration. One mL samples were spun down for 2 min at 16,100×*g*. The cells were then resuspended in 1 mL MRS-bile salts or low pH-MRS and vortexed, immediately after which 100 µL samples were taken for flow cytometry analysis. The remaining samples with MRS-bile salt or low pH-MRS were then incubated at 37 °C and new samples for survivability assays were taken every 30 min. The survival of the cells was checked with a flow cytometer (BD Accuri C6 plus flow cytometer with a BD C sampler (BD Biosciences, Franklin Lakes, NJ, USA) using a protocol described below.

### Flow cytometry

The flow cytometry protocol is based on the standard staining method of the Swiss Federal Institute of Aquatic Science and Technology, Eawag, Switzerland (Hammes et al. 2008) for analyzing the quality of drinking water. It makes use of the two different dyes SYBR^®^ Green I (Life Technologies) and Propidium Iodide (PI) (Sigma-Aldrich) for staining of the cells. The SYBR Green I penetrates all cells and binds selectively to double-stranded DNA (Zipper et al. 2004) and cells with compromised membranes are stained by PI that binds to DNA and RNA, whereas PI is excluded from cells with intact membranes due to the positive charge of PI (Shapiro 2003). Samples were diluted in PBS with the addition of 1 mM EDTA (Fluka) and 0.01% (v/v) Tween 20 (Sigma), to achieve a cell concentration of 1 × 10^3^–5 × 10^6^ cells/mL. For each sample a mix of 5 μL of 100X SYBR^®^ Green I (diluted in DMSO) and 1 μL PI (1 mg/mL) was used. The final volume of each sample was 506 μL. The samples were vortexed and then incubated in the dark for 15 min at 37 °C. After incubation, the samples were vortexed again and analyzed on the BD Accuri C6 plus flow cytometer with the fast fluid speed (flow rate of 66 μL/min), a sample volume of 50 μL and using a FL1-H and FL3-H threshold of 1000. Tubes with milli-Q-water were run between samples to rinse the system. The blue 488 nm laser was used with the optical filters FL1 (533 ± 30 nm) and FL3 (> 670 nm). The data were collected and analyzed using the BD Accuri C6 Plus software. A log scale density plot of FL1-H vs FL3-H was obtained to visualize the fluorescence of the dyes. Instrument settings and electronic gates were kept the same for all samples to achieve comparable data.

### Data analysis

A response surface methodology (RSM) was applied using a script previously written in Python 2.7 by the authors to process the experimental data. The resulting response surface and contours were graphed and fitted to the equation of a plane with intercept 0 assuming only the pure linear effect of these two factors on the survival rate since it was a screening design. The significance of the model coefficients was evaluated with the same script using the package Statsmodels.api (Python 2.7). The data of the post-stress tests were analyzed with ANOVA-single factor in Microsoft Office Excel 2007.

## Results

### Fermentation behavior at 1 L scale

**Fig. 1:**
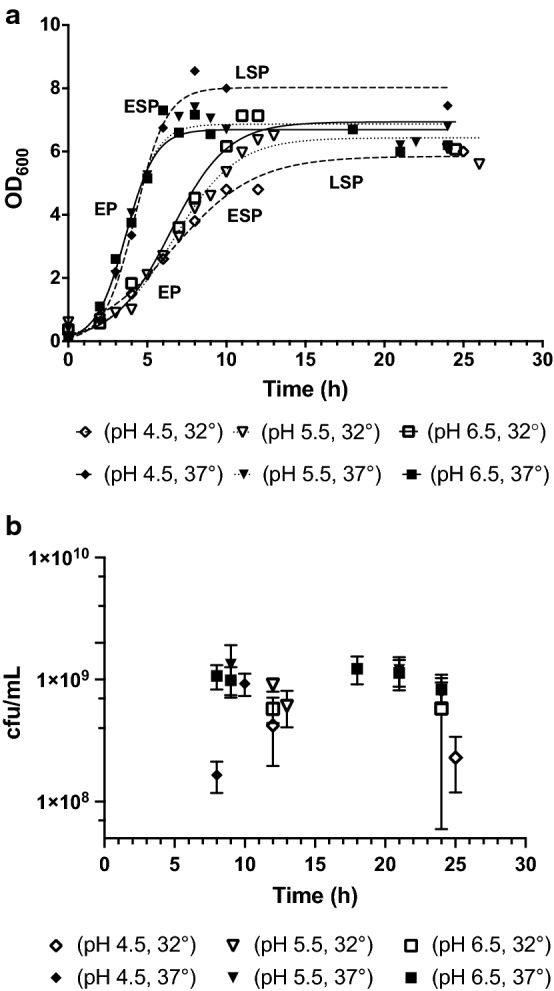
Fermentation of DSM 17938 during the 1-L scale under different conditions of pH and temperature. **a** Growth curves. **b** Cell concentration over time. The cells were cultured in MRS under anaerobic conditions and agitation speed 100 rpm. n = 2 replicates. *EP* exponential phase, *ESP* early stationary phase, *LSP* late stationary phase

The initial number of cells is an important variable to be considered when bacterial cells are preserved by desiccation methods (Morgan et al. 2006; García 2011), and most often the aim is to maximize the yield at the end of the fermentation (Alonso 2016). In addition, the physiological state of the bacterial cells is crucial to achieve high survival rates when drying. For instance, the change in unsaturated to saturated fatty acids ratio to keep the membrane fluidity, the active synthesis of stress proteins to protect cell structures, the expression of antioxidative defenses such as superoxide dismutase (SOD), the presence of compatible solutes, and the uptake of Mn^2+^ are among the mechanisms to prevent the desiccation damage (Potts et al. 2005; Garcia 2011). To evaluate the possible combined effects of pH and temperature on the biomass production of strain DSM 17938, the growth curves (Fig. 1a) and the growth phases were defined as follows: (i) exponential phase (between 2 and 8 h for cells cultivated at 32 °C, and between 2 and 5 h for cells cultivated at 37 °C), (ii) early stationary phase (between 10 and 12 h for cells cultivations at 32 °C, and between 7 and 9 h for cultivations at 37 °C), and (iii) late-stationary phase (after 15–16 h for cultures performed at 32 °C and after 9–10 h for cultivations at 37 °C). The specific growth rate (µ_MAX_) and generation time (t_g_) were estimated by fitting the growth curves to the logistic model (Peleg and Corradini 2011) (Table 1). For every temperature condition, the target pH was reached and controlled at different stages of the culture: (1) pH 6.5 was controlled since the beginning of the fermentation, (2) pH 5.5, was reached at exponential phase, (3) pH 4.5 was reached at early stationary phase (data not shown).

**Table 1:** Growth rate and generation time of *Lactobacillus reuteri* DSM 17938 cultured under different conditions

Experimental condition	μ_max_ (h^−1^)	t_g_ (h)
pH 4.5, 32 °C	0.51 ± 0.06	1.36 ± 0.16
pH 4.5, 37 °C	1.09 ± 0.22	0.64 ± 0.13
pH 5.5, 32 °C	0.55 ± 0.06	1.26 ± 0.14
pH 5.5, 37 °C	1.13 ± 0.21	0.61 ± 0.11
pH 6.5, 32 °C	0.59 ± 0.14	1.17 ± 0.28
pH 6.5, 37 °C	1.11 ± 0.23	0.62 ± 0.13

The fermentation was performed using MRS and different combinations of pH and temperature

The growth rate of the strain was twofold higher at 37 °C compared to 32 °C; moreover, the growth curves were similar at the three pH levels for every temperature in terms of growth rate and generation time (Table 1); although small differences were shown at pH 4.5 for both temperatures regarding OD_600_: at 32 °C the growth curve showed slightly lower absorbance values than at other pH, and in contrast, at 37 °C the absorbance values were slightly higher.

In addition, the cell viability of the cultures was monitored between 8 and 25 h. Even for the most severe culture conditions tested (pH 4.5 at late-stationary phase) the cell concentration reached between 10^8^ and 10^9^ cfu/mL and in all the variants, the cell viability was above 10^8^ cfu/mL (Fig. 1b).

### Effect of temperature and culture pH on freeze-drying survival rates

**Fig. 2:**
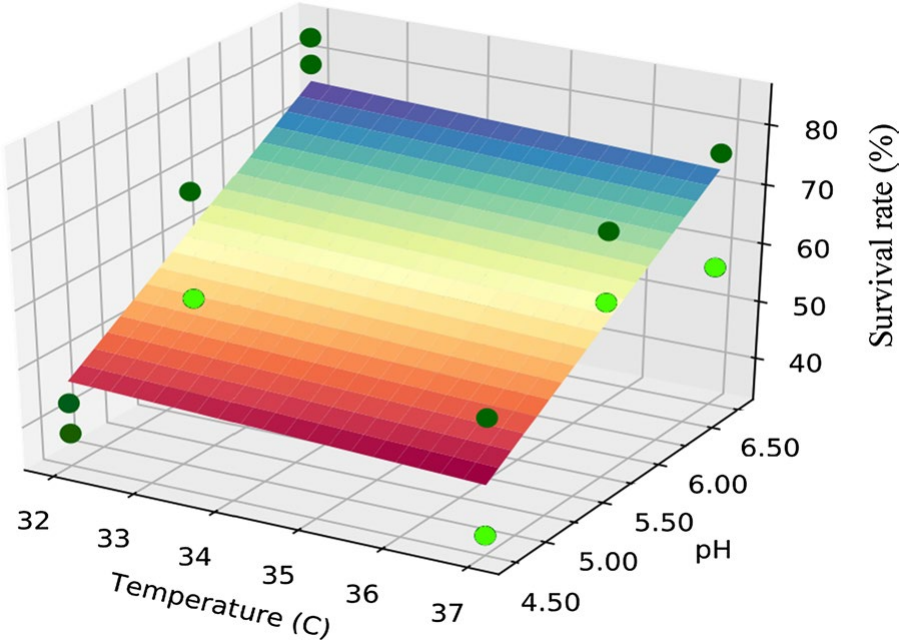
Freeze-drying survival rate of DSM 17938 as function of pH and temperature. A RSM was applied and the data were fitted to a linnear model. n = 2 replicates

To determine the impact of the culture variables pH and temperature on the freeze-drying survival of DSM 17938 cells cultured at two temperatures (32 and 37 °C) in combination with three pH levels (4.5, 5.5, and 6.5), a response surface methodology (RSM) was used (Fig. 2). No significant impact of temperature on the survival rates was found for the tested temperatures 32 to 37 °C (control) (P-value = 0.338). However, a highly significant impact of increased culture pH on the survival rate was found (P-value = 0.001) (Table 2). Some fluctuations were observed in the response (Fig. 2), which may be due to the treatment of the samples since they were stored at − 50 °C immediately after every freeze-drying process and rehydrated the next day in the morning at room temperature.

**Table 2:** Statistical analysis corresponding to the factorial design performed

Dependent variable: survival rate (%)	R-squared: 0.977
Method: least squares	Adj. R-squared: 0.972
Number of observations: 12	F-statistic: 209.9
Degrees of freedom residuals: 10	Probability (F-statistic): 6.82 × 10^−9^
Degrees of freedom: 2	
Coefficient for Temperature: − 0.534	Probability: 0.338
Coefficient for pH: 14.305	Probability: 0.001

A Python script was written for the analysis. Survival rate was the dependent variable, and pH and temperature the independent factors. The surface was fitted to a plane with intercept 0

### Post-stress tests survival rates

**Fig. 3:**
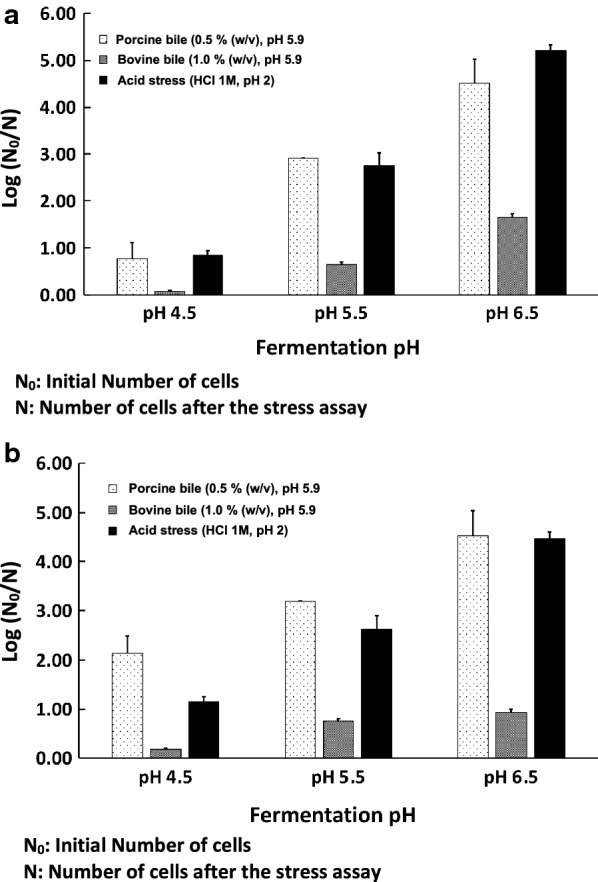
Stress survival tests on rehydrated freeze-dried DSM 17938 as function of pH and temperature. **a** Cells cultivated at 37 °C. **b** Cells cultivated at 32 °C

To investigate if fermentation conditions had any effect on tolerance to bile salts and low pH, stress survival tests on rehydrated freeze-dried cells were performed and analyzed using flow cytometry. Two different origins of bile salts were chosen. Bovine bile salts are known to be less inhibitory than that of porcine, the latter is similar to human bile salts (Grill et al. 2000). The two bile stress tests showed similar tendency in that cells cultured at higher pH possessed lower survival rates during these stress tests (P-value with porcine and bovine bile salts at 37 °C being 0.018 and 0.011, respectively) (Fig. 3a). The difference between the bile salt stress tests concerned only the inhibition capacity of the two different bile salts, i.e., 0.5% porcine bile killed more cells than 1% bovine bile.

The low pH stress tests led to lower stress survival rates when cells were grown at higher pH (P-value of 0.0005 at 37 °C) which was similar to what was observed with the bile salt test (Fig. 3a).

The same trends for the bile- and low-pH stress tests were observed at 32 °C (Fig. 3b) as for 37 °C, with P-values between 0.003 and 0.041.

## Discussion

This study was carried out to determine the effects of temperature and pH as growth parameters on the survivability of *L. reuteri* DSM 17938 to freeze-drying and to subsequent GIT conditions of rehydrated cells to mimic the process- and application stresses encountered by probiotic bacterial cells in both production and use. Different combinations of growth pH levels and temperatures were tested. The values of the specific growth rate (µ_MAX_) and generation time (t_g_) of the fermentations at 37 °C and pH 5.5 (Table 1) are in the range previously reported (Burgé et al. 2015; van Niel et al. 2017). In addition, since the growth rate and generation time were similar for every pH condition at the same temperature, it suggests that the growth of the DSM 17938 is not limited by pH when it grows without pH control until the fermentation reaches pH 4.5. Only the OD_600_ showed differences between the variants at the end of the culture, likely because of the auto-aggregation phenomenon which has been previously reported to be less extensive in another *L. reuteri* strain (TMW1.106) when cultivated in MRS and exposed to pH 4 (Walter et al. 2008).

The twofold increase in growth rate at 37 °C as compared to 32 °C irrespective of culture pH, reflects that the growth of DSM 17938 is temperature-sensitive in the narrow temperature interval investigated here. In addition, the OD_600_ at pH 4.5 behaved differently at the two temperatures. The higher absorbance observed at 37 °C might be related to a lower degree of auto-aggregation of the cells than observed at 32 °C. A similar trend has been observed in *L. johnsonii* CRL 1294 where the auto-aggregation percentage decreased from 76.7% to 67.8% when cultured in MRS at pH 5 at 30 °C and 44 °C, respectively (Juárez Tomás et al. 2005). The distribution of cell aggregates might therefore depend on the combination of pH and temperature. At higher temperature and lower pH, auto-aggregation seems to be less extensive for DSM 17938. The phenomenon of auto-aggregation should be evaluated in further studies since it is of importance for biofilm formation in the colonization of probiotic bacteria (Juárez Tomás et al. 2005; Leccese Terraf et al. 2014).

The viability of strain DSM 17938 remained above 10^8^ cfu/mL despite the exposure of the cells to an acidic environment (pH 4.5) (Fig. 1b). Similar behavior has been observed in the parental strain ATCC 55730 when exposed to more drastic conditions (pH 2.7) for one hour (Wall et al. 2007). It would be interesting in future work to test the survival of DSM 17938 under such drastic conditions and simultaneously perform intracellular pH assays to determine if cells manage to keep their pH homeostasis.

Preconditioning or pre-adaptation of cells during fermentation has notable impact on the survival rates during freeze-drying of probiotic bacteria (Shin et al. 2018; Anandharaj et al. 2017). For instance, the pre-adaptation by both heat and acid stress has shown to improve the freeze-drying survival rates in *Enterococcus faecium* HL7 (Shin et al. 2018). In our study, a 5 °C variation in culture temperature does not promote additional protection against desiccation by freeze-drying, whereas increased culture pH positively impacts the desiccation tolerance of this probiotic strain in the experimental interval tested (Table 2). Studies with other *Lactobacillus* species subjected to freeze-drying have shown that an increase of 8 °C in early stationary phase did not influence the freeze-drying survival rate. However, a decrease of 8 °C drastically reduced the freeze-drying survival of this strain as compared to when it was grown at 34 °C and pH 5.5 (Schoug et al. 2008). Regarding the impact of pH on freeze-drying survival, studies performed in *L. rhamnosus* GG revealed that cells harvested at late stationary phase grown under uncontrolled pH survived freeze-drying better than cells grown under controlled pH; however, the opposite effect was observed when cells were harvested in early stationary phase (Ampatzoglou et al. 2010).

In our study, the survival rates of DSM 17938 after freeze-drying are in the range published for other lactobacilli (Martos et al. 2007; Schoug et al. 2008; Liu et al. 2014). According to these results, formulation of the cells in 10% sucrose provides sufficient protection during freeze-drying to allow for a comparative study of growth condition effects on freeze-drying survival. Our findings differ from that reported by Palmfeldt and Hahn-Hägerdal (2000) for the parental strain ATCC 55730. According to these authors, the freeze-drying survival rate of the parental strain, as assessed by colony forming units, increases when the pH of the culture is decreased. However, their formulation differed since they used skim milk as lyoprotectant instead of sucrose which should be kept in mind when comparing the results. In a more recent study with *L. reuteri* I5007, Liu et al. (2014) have shown results similar to ours, with higher survival rates at pH 6.7 than at pH 4.7 as determined by viable cell counts. These researchers cultivated their strain in MRS and exposed it to various stress conditions, including pH stress (4.7 to 6.7), heat shock at 47 °C, and cold shock at 27 °C, from which they concluded that the highest survival rate was achieved when cells were subjected to pH 6.7 at late stationary phase. Therefore, culturing strain DSM 17938 at higher pH could be a useful option to improve survivability and therefore higher number of viable cells in the final product.

The pH of the fermentation culture influenced post-desiccation stresses (low-pH stress and bile salt stress) survival in an opposite manner as it did for freeze-drying survival per se. A lower culture pH enabled cells to survive exposure to low pH and bile salts better than for cells cultured at higher pH. Preconditioning through exposure to mild pH stress to boost survivability to a more severe pH shock has been observed for many other microorganisms (Skandamis et al. 2008; Cañamás et al. 2009). Proteomic studies with another *L. reuteri* strain (ATCC 23272) revealed which key proteins were overexpressed to adapt to these conditions, and included proteins related to transport, energy metabolism, biosynthesis of amino acids and nucleotides, and pH homeostasis (Lee et al. 2008a). Interestingly, similar key proteins were overexpressed in strain ATCC 23272 when exposed to purified bile salts (Lee et al. 2008b). This supports our observation that cells grown at lower pH have better survivability to bile salt exposure. In addition, similar finding has been reported for *L. reuteri* ATCC 55730, the parental strain of DSM 17938 (Whitehead et al., 2008). These authors found that genes implicated in protection to bile also protect against acid stress in *L. reuteri*, which could explain our results for freeze-dried DSM 17938.

For the fermentation temperature range studied herein (32 to 37 °C), no significant effect on the survivability in post-desiccation stress exposures was observed. Therefore, larger differences in temperature should be tested in future experiments, or alternatively, the biomass subjected to heat shock at sub-lethal temperatures to verify if significant differences are observed in freeze-drying survival rate.

As discussed above and as observed in other studies, preconditioning at low pH improves survivability during a pH and bile salt shock (Yadav and Shukla 2017; Chen et al. 2017). Moreover, the stress responses to an acidic pH have become better understood, but apparently these responses are detrimental for freeze-drying tolerance. Instead, the improvement of the latter emerges during growth at higher pH (around neutral) and might be related to a higher ratio of unsaturated fatty acids to saturated fatty acids in the cell membrane (Wang et al. 2005; Liu et al. 2014), which will be evaluated in further experiments, as well as the analysis of membrane fluidity by flow cytometry (Marielle and Sarrah 2017). Thus, responses do occur in the cells that are divergent and not completely streamlined for the series of stress events the *L. reuteri* DSM 17938 cells will have to undergo when produced and used. This demands that an appropriate solution for maximizing survivability over the whole life cycle from production to consumption is found: either (i) a compromise weighing in all the different encountered stress characteristics, or (ii) by providing the probiotic bacteria in capsules to avoid direct exposure to bile and acid in the GIT that would also allow for production of more freeze-drying tolerant biomass by growing at higher pH.

A subsequent comparative and functional proteomics and transcriptomics would be the appropriate tool to analyze how the cells in such a process become more tolerant to the freeze-drying stress.

In conclusion, culture pH in the range of 4.5–6.5 had a strong influence on freeze drying and post-desiccation stress survival whilst growth temperature in the range of 32–37 °C had no significant effects. Survivability to freeze drying is better for cells grown at higher pH, whereas survivability to GIT conditions is better for cells grown at lower pH. Culturing strain DSM 17938 at higher pH values could be a useful option to improve survivability and therefore higher viable cell numbers in the final product, however an optimization of the process should be performed to improve the resistance of these cells to the acidic environment in the stomach.

## Data Availability

All data and material are available without any restriction.
